# Comment on: Significant association of serum H. pylori IgG antibody titer with kidney function in renal transplanted patients

**DOI:** 10.12861/jrip.2013.04

**Published:** 2013-03-01

**Authors:** Mohamad Reza Tamadon

**Affiliations:** ^1^Department of Internal Medicine, Semnan University of Medical Sciences, Semnan, Iran

**Keywords:** *Helicobacter pylori*, Kidney transplantation, Renal function

**Implication for health policy/practice/research/medical education:**

*Helicobacter pylori* infection remains one of the most challenging infectious diseases causing high mortality and morbidity. The prevalence of infection varies greatly between industrial and developing countries as well as between different geographic areas.

*Helicobacter pylori *(*H. Pylori*) infection remains one of the most challenging infectious diseases causing high mortality and morbidity. The prevalence of infection varies greatly between industrial and developing countries as well as between different geographic areas (1-3). In the past years, several diagnostic methods is provided for the detection of *H. Pylori *infection which are generally divided into two categories: direct methods (invasive) and indirect (non-invasive). Direct methods involve biopsies of ulcer margins by endoscopy and microscopic examination, endoscopy with phenols staining, rapid urease test, and histological specimen culture and polymerase chain reaction. Indirect methods include antibody serology test, stool antigen test, urea breath test, examining of teeth plaque, urine and gastric juice examination (4). Today, pay more attention to the noninvasive diagnostic tests (5,6). Some studies reported the value of noninvasive diagnostic tests (7,8).In the present study relationship between *H. Pylori *IgG antibodies with renal function assessed in patients who underwent kidney transplantation. A similar study in hemodialysis patients previously conducted, the results indicate the value of these tests (7). With regard to the specific situation of kidney transplant patients specially receiving the immunosuppressive drugs for a long period, one of the main matters that should be considered is the *H. Pylori *infection. In this study, there is a relationship between the *H. Pylori *IgG antibodies titer and renal function so that with regard to the specific situation of these patients we can eradicate *H.Pylori*infection and prevent from occurrence of any serious complications. This is a very valuable point in this study.

## Author’s contribution

MRT is the single author of the manuscript.

## Conflict of interests

The author declared no competing interests.

## Ethical considerations

Ethical issues (including plagiarism, data fabrication, double publication) have been completely observed by the author.

## Funding/Support

None declared.

## References

[1] Fock KM, Ang TL (2010). Epidemiology of Helicobacter pylori infection and gastric cancer in Asia. J Gastroenterol Hepatol.

[2] Graham DY, Adam E, Reddy GT, Agarwal JP, Agarwal R, Evans DJ Jr (1991). Seroepidemiology of Helicobacter pylori infection in IndiaComparison of developing and developed countries. Dig Dis Sci.

[3] Taylor DN, Blaser MJ (1991). The epidemiology of Helicobacter pylori infection. Epidemiol Rev.

[4] Kim N, Kim JJ, Choe YH, Kim HS, Kim JI, Chung IS (2009). Diagnosis and treatment guidelines for Helicobacter pylori infection in Korea. Korean J Gastroenterol.

[5] Qujeq D, Savadkohi Sh (2006). Non- invasive assay for diagnosis of Helicobacter pylori. Hakim.

[6] Cirak MY, Akyön Y, Mégraud F (2007). Diagnosis of Helicobacter pylori. Helicobacter.

[7] Tamadon MR, Saberi Far M, Soleimani A, Ghorbani R, Semnani V, Malek F, Malek M (2013). Evaluation of noninvasive tests for diagnosis of Helicobacter pylori infection in hemodialysis patients. J Nephropathology.

[8] Redeen S, Petersson F, Tornkrantz E, Levander H, Mardh E, Borch K (2011). Reliability of Diagnostic Tests for Helicobacter pylori Infection. Gastroenterol Res Pract.

